# Reactivation of Multidrug-Resistant HSV-1 in a Post–Allogenic Hematopoietic Stem Cell Transplant Patient: Dynamic Detection of the Rare A605V Mutation by Next-Generation Sequencing

**DOI:** 10.1093/ofid/ofae250

**Published:** 2024-05-03

**Authors:** Shuxuan Zheng, Lidewij W Rümke, Bruno Tello Rubio, Malbert R C Rogers, Geerte L van Sluis, Jürgen H E Kuball, Annelies Riezebos-Brilman, Robert Jan Lebbink, Frans M Verduyn Lunel

**Affiliations:** Department of Medical Microbiology, UMC Utrecht, Utrecht, The Netherlands; Department of Medical Microbiology, UMC Utrecht, Utrecht, The Netherlands; Department of Medical Microbiology, UMC Utrecht, Utrecht, The Netherlands; Department of Medical Microbiology, UMC Utrecht, Utrecht, The Netherlands; Department of Haematology UMC Utrecht, Utrecht, The Netherlands; Department of Haematology UMC Utrecht, Utrecht, The Netherlands; Department of Medical Microbiology, UMC Utrecht, Utrecht, The Netherlands; Department of Medical Microbiology, UMC Utrecht, Utrecht, The Netherlands; Department of Medical Microbiology, UMC Utrecht, Utrecht, The Netherlands

**Keywords:** aciclovir, foscarnet, HSV-1, multidrug resistance, stem cell transplantation

## Abstract

We present an immunocompromised patient with a multiresistant herpes simplex virus–1 reactivation with a rare mutation (A605V) in the viral DNA polymerase gene. Next-generation sequencing suggests the presence of multiple drug-resistant strains before treatment and altered ratios during treatment, affecting the clinical response to aciclovir and foscarnet.

Hematopoietic stem cell transplant (HSCT) recipients face a substantial risk of severe herpes simplex virus (HSV) reactivation [1]. Drugs of choice for prophylaxis and therapy are nucleoside analogues aciclovir (ACV) and its prodrug valaciclovir (ValACV) [2]. Resistance to ACV and ValACV often coincides with cross-resistance to other nucleoside analogues such as penciclovir and its prodrug famciclovir [3]. In infections refractory to nucleoside analogues, other therapeutic options are the more toxic agents: foscarnet (FOS; a pyrophosphate analogue) and cidofovir (CDV; a nucleotide analogue) [2, 4]. Resistance to HSV antivirals can be attributed to changes in the viral enzyme thymidine kinase (TK; encoded by the UL23 gene) and viral DNA polymerase (DNA *pol*, encoded by the UL30 gene) [4, 5]. To exert activity, ACV first needs to be phosphorylated by viral TK and cellular kinases before targeting DNA *pol*, whereas FOS directly inhibits viral DNA *pol* (UL30) [6]. In contrast to TK, functional DNA *pol* is essential for viral replication, and therefore DNA *pol* mutations in clinical isolates rarely occur [4, 6, 7]. Drug-resistant mutations in HSV-1 can be identified at the genetic level using various techniques, including conventional Sanger sequencing and, more recently, next-generation sequencing (NGS) [2, 8]. The latter has the additional advantage of enhanced sensitivity to detect minor variants in mixed populations [8]. In this study, we present an HSCT patient experiencing reactivation of HSV-1 that is resistant to ACV and FOS treatment. This resistance was attributed to a mutation in the TK gene (R281STOP, associated with ACV resistance) as well as a less common mutation in the DNA *pol* gene (A605V, associated with ACV and FOS resistance). Notably, these mutations were detected at distinct time points during the course of treatment by means of NGS to monitor the kinetics of the infection. Moreover, we conducted phenotypic resistance testing to further elucidate the properties of the infrequent A605V mutation.

## CASE REPORT

**Figure 1.**
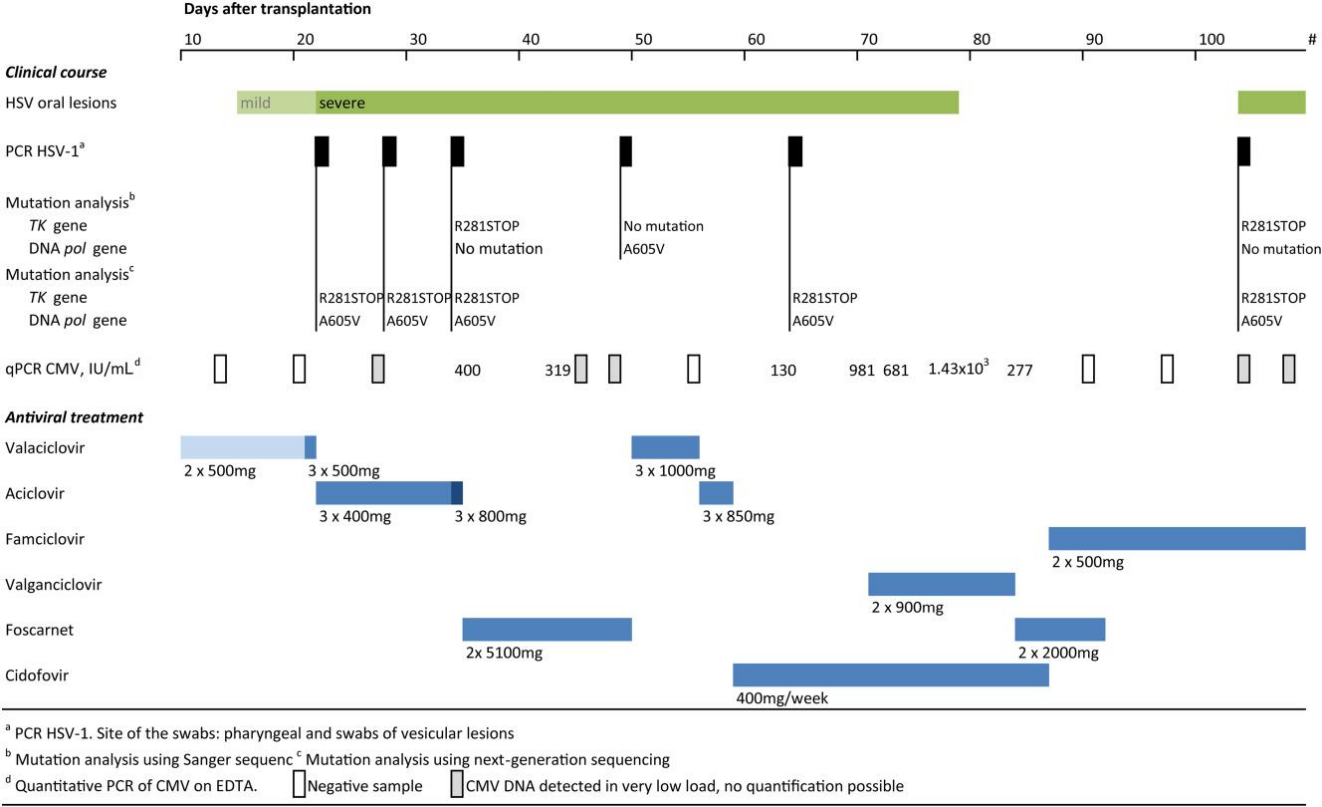
Overview of the clinical course and treatment of multidrug-resistant herpes simplex virus 1 reactivation in a post–allogenic hematopoietic stem cell transplant patient. The patient was treated for HSV-1 stomatitis as follows: day 22 after transplantation, positive HSV-1 real-time PCR, treatment with valaciclovir initiated; day 34, switch to aciclovir due to disease progression; day 36, switch to foscarnet due to concurrent CMV reactivation and the finding of an acyclovir-associated resistance mutation (R281STOP) in the *TK* gene; day 49, detection of mutation in the DNA *pol* gene (A605V) associated with resistance to aciclovir and foscarnet, subsequent switch to cidofovir at day 59 until resolution of HSV-1 stomatitis; day 104, recurrence of herpetic lesions, R281STOP mutation detected, A605V mutation not detected. Abbreviations: CMV, cytomegalovirus; DNA *pol*, DNA polymerase; HSV, herpes simplex virus; PCR, polymerase chain reaction; qPCR, quantitative PCR; TK, thymidine kinase.

We present a 61-year-old male with chronic lymphocytic leukaemia who underwent a nonmyeloablative allogeneic cord blood stem cell transplantation (allo-HSCT). Before transplantation, the patient was HSV-1 seropositive. ValACV (500 mg, 2 times a day orally) was prescribed as varicella zoster and HSV prophylaxis 1 week before allo-HSCT. At day 22, a swab of oral ulcerations tested positive for HSV-1 by real-time polymerase chain reaction (PCR), and ValACV treatment (500 mg, 3 times a day orally) was initiated and after 1 day modified to intravenous ACV (5 mg/kg, thrice daily) (Figure 1). Due to persistent herpetic stomatitis, intravenous ACV was increased to 10 mg/kg thrice daily on day 34. This adjustment was followed by a transition to intravenous FOS on day 36 due to the identification of a mutation in the HSV-1 TK gene, specifically a premature stop codon at amino acid position 281 (R281STOP), which coincided with a concurrent reactivation of cytomegalovirus (CMV). The R281STOP mutation is associated with reduced susceptibility to ACV, brivudine, and famciclovir in vitro [2]. Despite the administration of FOS, there was no discernible improvement in the herpetic lesions. Consequently, the treatment was reverted to ValACV. Subsequent genotypic resistance analysis of a patient sample collected at day 49 unveiled a rare mutation in the DNA *pol* gene (A605V). This mutation is known to confer resistance to both ACV (ACV^r^) and FOS (FOS^r^) [2]. Following this discovery, treatment with ACV was switched to intravenous CDV administration. Valganciclovir was added to the antiviral regimen due to persistent CMV reactivation. After 3 weeks on CDV, the herpetic lesions improved and CDV was switched to oral famciclovir to prevent varicella-zoster virus (VZV) and HSV reactivation. As there were no other oral alternatives for prophylaxis, famciclovir was prescribed despite the possibility of cross-resistance of (latent) HSV-1 to famciclovir based on the previously detected R281STOP mutation. On day 104, the oral herpetic lesions recurred, and repeated genotypic analysis revealed re-emergence of the R281STOP mutation, but not the A605V mutation. Unfortunately, the patient died afterwards from complications of graft-vs-host disease.

## RESULTS

Based on the dynamic occurrence of the 2 drug resistance mutations during treatment, we hypothesized that multiple drug-resistant HSV-1 variants might be present in the patient. To validate this hypothesis, we performed NGS on patient samples obtained at days 22, 28, 34, 64, and 104 post-allo-HSCT. The regions of the viral TK and *pol* genes that include the R281STOP and A605V mutations were PCR-amplified and subjected to Illumina sequencing. Upon aligning the sequences to the reference HSV-1 strain (GenBank accession number: JQ673480), both mutations were identified. Notably, at day 22, post-transplantation sequences derived from both TK_R281STOP- and DNA *pol*_A605V-containing viruses were simultaneously present in, respectively, 39.94% and 0.13% of the sequences (Table 1). At that time point, antiviral therapy had not yet been initiated. Under antiviral pressure in vivo, the ratio of HSV-1 strains carrying these mutations changed dramatically over time.

**Table 1.** Summary of the Results Obtained From Breseq

Days After Transplantation	*TK*_R281STOP, %	Total Reads	DNA *Pol*_A605V, %	Total Reads
22	39.94	145 163	0.13	373 554
28	98.77	257 124	0.83	396 946
34	94.76	358 015	0.66	372 147
64	1.09	282 560	31.21	632 980
104	99.75	400 428	0.30	413 501

The mutation percentages (*TK*_R281STOP and DNA_*pol*_A605 V) were calculated by dividing the total amount of mapped reads containing the specific alternative base at the specific position by the total amount of reads mapped to that position (Total Reads). TK and DNA *pol* fragments were amplified in different runs causing differences in total number of reads (Supplementary Data).

In vitro phenotypic characterization was performed to validate the phenotypic antiviral resistance profile of our patient-derived HSV-1 isolate harboring the DNA *pol*_A605V mutation. Our analysis confirmed resistance of the HSV-1 DNA *pol*_A605V isolate to FOS with concentrations exceeding the known effective threshold of ∼100 µg/mL (Supplementary Figure 1a). At higher concentrations (600–1200 µg/mL), FOS was able to inhibit virus replication, thereby abrogating virus-induced cell death. We also investigated resistance of the HSV-1 DNA *pol*_A605V variant to ACV (Supplementary Figure 1b). Our results indicate that the clinical HSV-1 DNA *pol*_A605V isolate is resistant to ACV, with an ACV IC_50_ of 4.54 µg/mL. This finding aligns with the established definition of ACV resistance, where IC_50_ values >2 µg/mL are generally considered a cutoff for ACV resistance [9]. The ACV^r^ and FOS^r^ phenotypes of the DNA *pol*_A605V-harboring isolate corresponded with clinical failure on ACV and FOS in our patient.

## DISCUSSION

Here, we report the first clinical case of an immunosuppressed patient with HSV-1 reactivation after allo-HSCT with 2 concurrent antiviral resistance mutations (TK_R281STOP and DNA *pol*_A605V). Further characterization using NGS revealed that these mutations were presented with dynamic ratios during antiviral treatment. This emphasizes that HSV-1 strains carrying resistance mutations can persist in latent HSV-1 and reappear under antiviral pressure.

The rare A605V mutation in the DNA *pol* gene has been reported occasionally [4, 10–12]. The mutation is associated with in vitro resistance to both ACV and FOS [2], which corresponds with the in vivo clinical failure on ACV and FOS observed in our patient. During follow-up, the A605 V mutation became undetectable by Sanger sequencing of the DNA *pol* gene in the absence of ACV and FOS, whereas the premature stopcodon mutation on position 281 in the TK gene re-appeared on ACV treatment. NGS detected the presence of both the DNA *pol*_A605V and TK_R281STOP mutations before antiviral treatment and indicates a mixed infection with the 2 mutations throughout the post-allo-HSCT period. Previous research has characterized HSV-1 strains with mutations in the essential DNA *pol* gene as having attenuated growth. TK activity is not essential for viral replication in replicating cells, such as cells used in standard cell culture [13], but is essential for viral replication in nonreplicating cells. As both the DNA *pol*_A605V variant and the TK_R281STOP variant retain replicative capacity [14], albeit at a reduced level, the possibility exists that both mutants established latency in nonreplicating cells such as neurons, which may explain the existence of both mutants before antiviral pressure. Different viral growth capacity of the HSV-1 resistance mutation–carrying strains may have contributed to the dynamic ratios of the 2 mutations, with the DNA *pol*_A605V mutant possibly exhibiting lower replicating capacity than the TK_R281STOP mutant. Thus, the 2 mutants showed compatible cooccurrence on antiviral treatment.

A diagnostic swab from the oral mucosa Is not necessarily representative of all variants that are present in a swarm of viruses in an immunocompromised person with extensive lesions.

Sanger sequencing could have missed the variant with the R281STOP mutation obtained while the patient was receiving FOS. It can also not be excluded that HSV strains may differ from each other between the anatomical locations within the same patient, though the latter seems of minor importance as clinically significant lesions only existed in the oral mucosa.

Our findings highlight that multiple HSV-1 strains carrying different resistance-associated mutations can persist in a patient. The utilization of NGS genotyping on clinical samples is a potent method for detection of low-abundant strains carrying drug resistance mutations and facilitates swift adjustments to effective treatment strategies.

## Supplementary Material

ofae250_Supplementary_Data

## Supplementary Data

Supplementary materials are available at *Open Forum Infectious Diseases* online. Consisting of data provided by the authors to benefit the reader, the posted materials are not copyedited and are the sole responsibility of the authors, so questions or comments should be addressed to the corresponding author.
