# Correction to: Reactivation of Multidrug-Resistant HSV-1 in a Post–Allogenic Hematopoietic Stem Cell Transplant Patient: Dynamic Detection of the Rare A605V Mutation by Next-Generation Sequencing

**DOI:** 10.1093/ofid/ofae373

**Published:** 2024-07-05

This is a correction to: Shuxuan Zheng, Lidewij W Rümke, Bruno Tello Rubio, Malbert R C Rogers, Geerte L van Sluis, Jürgen H E Kuball, Annelies Riezebos-Brilman, Robert Jan Lebbink, Frans M Verduyn Lunel, Reactivation of Multidrug-Resistant HSV-1 in a Post–Allogenic Hematopoietic Stem Cell Transplant Patient: Dynamic Detection of the Rare A605V Mutation by Next-Generation Sequencing, *Open Forum Infectious Diseases*, Volume 11, Issue 5, May 2024, https://doi.org/10.1093/ofid/ofae250.

A typographic error has been corrected in author Lidewij W Rümke's name.

