# Supplementary material for: Reactivation of Multidrug-Resistant HSV-1 in a Post–Allogenic Hematopoietic Stem Cell Transplant Patient: Dynamic Detection of the Rare A605V Mutation by Next-Generation Sequencing
Source: Open Forum Infect Dis. 2024 May 3;11(5):ofae250. doi: 10.1093/ofid/ofae250 (PMC11127484; doi:10.1093/ofid/ofae250)
Supplement: ofae250_Supplementary_Data [file ofae250_supplementary_data.docx]

SUPPLEMENTARY INFORMATION
**Reactivation of Multidrug-Resistant HSV-1 in a Post-Allogenic Hematopoietic Stem Cell Transplant Patient: Dynamic detection of the rare A605V mutation by next generation sequencing**

S. Zheng^1*^, L.W. Rümke^1*^, B. Tello Rubio^1^, M.R.C. Rogers^1^, G.L. van Sluis^2^, J.H.E. Kuball^2^, A. Riezebos-Brilman^1^, R.J. Lebbink^1^, F.M. Verduyn Lunel^1‡^

*^1^Department of Medical Microbiology, UMC Utrecht, Utrecht, The Netherlands
^2^Department of Haematology UMC Utrecht, Utrecht, The Netherlands*

**Equal contribution*

# ^‡^Corresponding author: [F.M.VerduynLunel@umcutrecht.nl](mailto:F.M.VerduynLunel@umcutrecht.nl)

# MATERIALS AND METHODS

## Cells

African green monkey kidney (Vero) cells were purchased from ATCC (American Type Culture Collection) (Rockville, USA). Cells were grown in DMEM (Dulbecco’s Modified Eagle Medium) (LonzaAG, Switzerland) supplemented with L-glutamine, penicillin-streptomycin and 10% fetal calf serum (FCS) at 37°C in a humidified incubator containing 5% CO_2_.

## Viruses

Clinical isolates of HSV-1 were isolated from sequential swabs of oral ulcerations collected on day 22, 34, 49, 64 and 104 post transplantation. The original swab media were found to be HSV-1 positive by real-time PCR analysis. Clinical isolates were further cultured in Vero cells. An eGFP-encoding laboratory strain was kindly provided by Prof. Peter O’ Hare (Imperial College, London, UK). The lab strain carries a VP16-eGFP recombinant protein which was engineered in HSV-1 strain 17. HSV-1-eGFP strain was propagated in Vero E6 cells using standard culturing and harvest techniques and viral titer was determined by plaque assay as described previously (1).

## Antiviral compounds

Acycloguanosine (Aciclovir, ACV) (Sigma-Aldrich, GmbH) and Foscarnet sodium (Bio-Connect, the Netherlands) were used as the antiviral compounds for the *in vitro* phenotypic characterization of HSV-1 clinical isolate and recombinant viruses.

## Virus purification by plaque assay

For cloning viruses, HSV-1 plaque assays were performed in triplicates for each biological sample. 200,000 Vero cells were seeded in a 12-well plate well in complete DMEM and infected the following day with various dilutions of supernatant harvested from HSV-1 infected Vero cells. After 2 hours incubation, cells were overlaid with 0.5% agarose (Seakem LE Agarose, Lonza AG, Switzerland) solution in complete DMEM and cultured for 3 days to allow the formation of plaques to occur. Single plaque that was isolated from other plaques were collected with a tip and transferred to prepared monolayers of the Vero cells for further culture. Subsequently, the plaque assay procedure was repeated twice to obtain a pure viral clone. Low-passage clonal HSV-1 strains were propagated (<5 passages) in Vero cells. Sequences of clonal viruses were further verified by Sanger sequencing.

## Genotypic characterization by Sanger sequencing of TK and DNA *pol* genes

The genotypic analysis of resistance of HSV-1 was carried out by the PCR amplification of DNA fragments of the viral TK and DNA pol genes followed by Sanger DNA sequencing. Viral DNA was isolated from virus supernatant using the Quick-gDNA™ MiniPrep kit (BaseClear B.V. the Netherlands). Proofreading Phusion DNA polymerase (Thermo Scientific) was used to amplify full-length of TK, whereas Expand High Fidelity DNA polymerase enzyme (Sigma-Aldrich, Merck) was used to amplify full-length of DNA *pol*. Primers for amplifying full length TK and DNA pol are listed in Supplementary Table S1. PCR products were purified from gel using the Gene-Jet PCR purification kit (Thermo Scientific), and subjected to Sanger sequencing (Macrogene Inc., the Netherlands). Sequencing primers are listed in Supplementary Table S1. Nucleotide sequence analysis was carried out using SnapGene V5.2 software (from Insightful Science; available at snapgene.com), alignment was performed by comparing with published sequences of the reference strain HSV-1 strain 17.

## Phenotypic characterization using plaque reduction assay

Antiviral testing for ACV and foscarnet (FOS) resistance was performed using a plaque reduction assay (PRA) in 12-well plates with Vero cells. Vero cells were seeded at density of 200,000 the day prior to infection. Next, cells were infected with clonal HSV-1 clinical mutants and HSV-1-eGFP at an MOI of 0.01. To assess the phenotypic drug response, ACV or FOS was added at the time of HSV-1 infection at various concentration (0, 10, 20, 40, 60, 80, 100 μM or 0, 300, 600, 1200 ng/ml, respectively). Cells were infected with viruses in the absence of ACV served as positive control, and uninfected cells served as negative control. Triplicates were included for each ACV or FOS concentration. Plates were incubated at 37 °C in a humidified air containing 5% CO2. After 3 days, cells were fixed overnight at room temperature with 3% formaldehyde and stained with 0.5% crystal violet. After 3 washes with water, phenotypic results are visible under naked eye.

**Next generation sequencing**

PCR was performed directly on patient samples (swabs of the oral ulcerations) at various time points (day 22, 28, 34, 64, 104) after transplantation by amplifying 283 bp and 334 bp fragments corresponding to the TK and DNA *pol* of interest. Sample preparation for next-generation sequencing involved two consecutive PCR reactions, referred to as PCR_overhang and PCR_index, for the addition of overhang and index oligos, respectively. Overhang sequences were added to the 5’ and 3’ end of the amplified viral DNA fragment of interest. These overhangs serve as binding sites for unique index oligos that are added in the subsequent PCR reaction. (see Supplementary Table 1), which are then pooled together. Sequencing was performed on the Illumina NextSeq500 platform (Illumina, Inc), generating paired-end 150 bp reads.

**Next-generation sequencing data analysis**

Paired-end sequencing reads were trimmed using Trimmomatic (2) with the following settings: ‘PE SLIDINGWINDOW:4:20 MINLEN:25 ILLUMINACLIP: NexteraPE-PE.fa:2:40:15’. The remaining trimmed reads were then used as input for Breseq (3) to call for mutations in the samples using the .gbk file of HSV-1 FOS (GenBank accession no. JQ673480) as the reference genome. The flags ‘--predict-polymorphisms --polymorphism-frequency-cutoff 0.0001’ were added to the Breseq execution command to specify for a mutation frequency threshold of 0.01%.

# REFERENCES

1. van Diemen FR, Kruse EM, Hooykaas MJ, Bruggeling CE, Schürch AC, van Ham PM, et al. CRISPR/Cas9-Mediated Genome Editing of Herpesviruses Limits Productive and Latent Infections. PLoS Pathog. 2016;12(6):e1005701.

2. Bolger AM, Lohse M, Usadel B. Trimmomatic: a flexible trimmer for Illumina sequence data. Bioinformatics. 2014;30(15):2114-20.

3. Deatherage DE, Barrick JE. Identification of mutations in laboratory-evolved microbes from next-generation sequencing data using breseq. Methods Mol Biol. 2014;1151:165-88.

4. Schneider CA, Rasband WS, Eliceiri KW. NIH Image to ImageJ: 25 years of image analysis. Nature Methods. 2012;9(7):671-5.


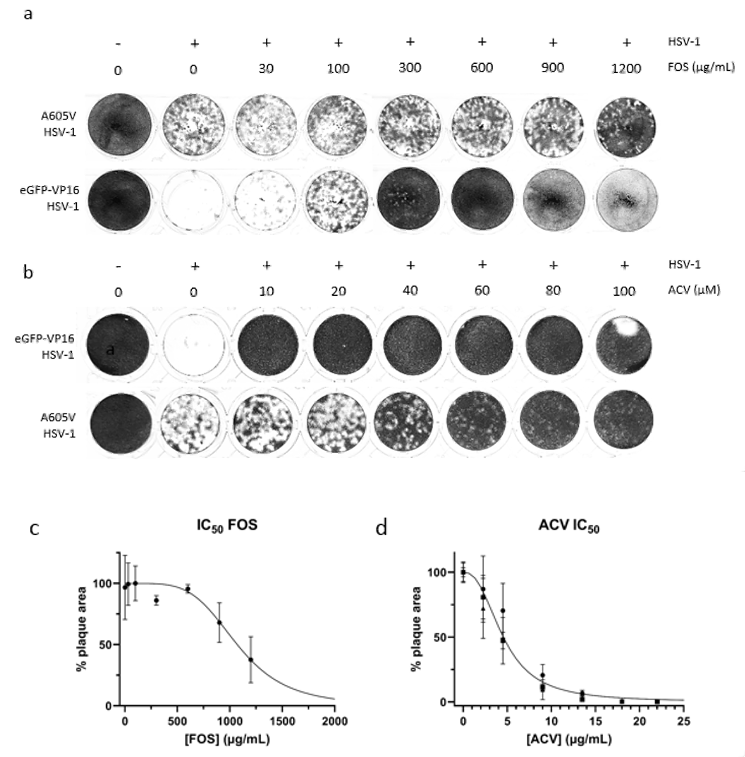


**Supplementary Figure 1.**

**Phenotypic charactering of resistance to ACV and FOS in HSV-1 DNA *pol*_A605V variant**

1. Visualization of plaque reduction assay (PRA). HSV-1 DNA *pol*_A605V variant and HSV-1 eGFP strain were cultured in Vero cells in the presence of indicated concentrations of foscarnet (FOS; 0, 30, 100, 300, 600, 900, 1200 μg/ml). After 6 days, cells were fixed and stained with crystal violet to visualize surviving cells. Negative control: Vero cells in absent of HSV-1 and FOS (0 μg/ml). Positive control: Vero cells in the presence of HSV-1 (MOI 0,01) only.
2. Same as in (a), yet aciclovir (ACV; 0, 10, 20, 40, 60, 80, 100 μM) was used instead of FOS.
3. FOS IC_50_ was calculated based on the results of a single PRA. HSV-1 DNA *pol*_A605V FOS IC_50_ = 1071 µg/mL (R2 = 0.6785). IC_50_ >100 µg/mL is associated with FOS resistance. Predicted model: curve fitting for FOS IC_50_ was achieved by plotting the obtained IC_50_ equation for prediction of lower % plaque are values. Plaque reduction % was obtained by ImageJ (4) of triplicate wells; plaque formation in the absence of drug treatment condition was used as a baseline to determine 100% of the plaque area.
4. ACV IC_50_ was calculated based on results from three independent PRAs (n=3) in which Vero cells were infected with HSV-1 DNA *pol*_A605V at an MOI of 0.1. DNA *pol*_A605V ACV IC_50_ = 4.54 µg/mL (R2 = 0.9293). IC_50_ >2 µg/mL is associated with ACV resistance.


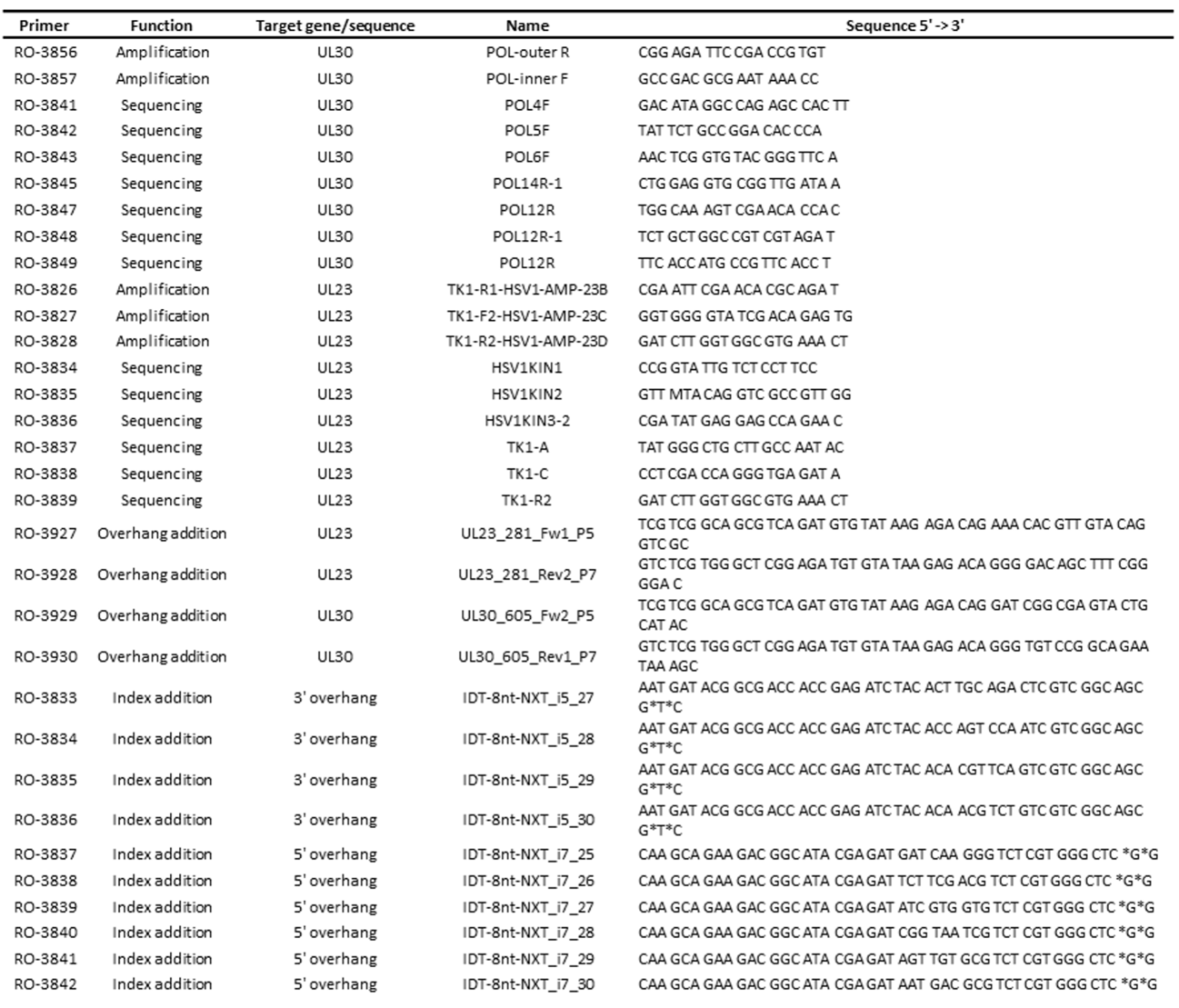


**Supplementary Table 1.**

**Primers used for PCR assays carried out throughout the genotypic analysis.**
